# Body-Efficacy Expectation: Assessment of Beliefs concerning Bodily Coping Capabilities with a Five-Item Scale

**DOI:** 10.1155/2013/152727

**Published:** 2013-11-07

**Authors:** Lena Schützler, Claudia M. Witt

**Affiliations:** Institute for Social Medicine, Epidemiology and Health Economics, Charité—Universitätsmedizin Berlin, 10098 Berlin, Germany

## Abstract

*Background*. Expectancies regarding a treatment play an important role in recovery as has been shown in placebo research. The role of expectations regarding the bodily capability to overcome illness is less investigated although in complementary and alternative medicine (CAM) such capability is the target of interventions. We introduced a new construct, body-efficacy expectation, defined as the conviction that one's body is able to deal with health-threatening factors by itself, and developed and validated a scale for its measurement. *Methods*. The scale was developed following expert recommendations. Using online survey data from 1054 participants an exploratory factor analysis was conducted and psychometric properties of the scale were examined (item characteristics, reliability, and validity). *Results*. The exploratory factor analysis yielded a one-factor solution explaining 51.96% of total variance (Cronbach's **α** = 0.77). One of the originally six items was removed due to poor item characteristics. Correlations with several validation measures were in line with the theoretical background of the construct. Most importantly, participants with better general health showed higher body-efficacy expectation than participants with poorer health status. *Conclusions*. Further studies confirming the factor structure and using clinical samples are recommended. Also, the relations with the appraisal of CAM and CAM use warrant further research.

## 1. Introduction

Expectations play a crucial role in the maintenance of health and recovery. This has especially been highlighted in placebo research. Expectations can be regarded as the core element of the placebo effect [1] which is also reflected in its NBCI's MeSH term databank definition as “an effect usually, but not necessarily, beneficial that is attributable to an expectation that the regimen will have an effect; that is, the effect is due to the power of suggestion.” But not only can one have expectations regarding the effectiveness of a treatment, but also can expectations concern the capability of one's body to overcome an illness, or to maintain health. Such expectancies are especially regarded in complementary and alternative medicine (CAM) since self-regulation is described as one aspect of CAM's anthropology [2]. Most forms of CAM assume a self-regulating force of the body, for example, the Qi in Traditional Chinese Medicine [3]. Illness is understood as an imbalance or weakened state of this force which therefore has to be rebalanced or strengthened by the treatment. In that way, expectancies regarding the effectiveness of a CAM treatment are subsequently also expectancies concerning the self-healing capacities of the body. In case they show effects similar to other placebo/expectancies effects [4, 5], they might be a large part of the effect of CAM treatments. 

In order to evaluate the positive influence on treatment outcome that beliefs concerning one's bodily capability might have and their role in CAM, first one needs a clear definition of such beliefs and a reliable and valid measure for their assessment. 

In developing both we were guided by scale development processes and research in the field of self-efficacy expectation. Self-efficacy expectation is defined as “the conviction that one can successfully execute the behaviour required to produce the outcomes” [6] and has proven to be an important construct in health psychology. Domain-specific self-efficacy expectations (i.e., convictions concerning narrowly defined behaviors as regularly brushing one's teeth or refraining from drinking) have been shown to be important predictors for changes in health behavior, coping with illness, or recovery [7–9]. General self-efficacy expectation, that is, the conviction that one is able to cope with demanding and/or novel situations in general [10], has been found to correlate with a variety of measures concerning wellbeing and coping [11–13]. Although the body's performance when restoring or maintaining health differs from such actively executable behaviors that are the subject of self-efficacy expectations we found that both issues resemble each other. We therefore called the beliefs about one's bodily capability *body-efficacy expectation* (BEE) and defined them as the conviction that one's body is able to heal and take care of itself by dealing with pathogens and other health-threatening demands on its own. Since Bandura [6] describes performance accomplishments as the most important source for the manifestation of self-efficacy expectations, one can assume that someone who experiences a “capable body”; that is, someone who is rarely ill or overcomes illness quickly has higher BEE than someone of bad general health. Also, someone who experienced, for example, in CAM treatment, that his/her condition can improve without the use of an “invasive” treatment (e.g., antibiotics) can be assumed to have higher BEE than someone without that experience. In fact, CAM users report about the treatment-induced experience that the body is able to heal itself [14, 15]. 

Having thus defined BEE, we aimed to develop a reliable and valid measure, investigate its factor structure and reliability, and in the validation process evaluate its relation with other health-related measures, namely, general and current health status (assessed with various measures), physical fitness, general self-efficacy, internal health-locus of control, and body care. In addition, we were interested in relations with CAM and/or conventional medicine usage, that is, appraisal of CAM, medication usage, and kinds of health-care professionals consulted.

## 2. Methods

### 2.1. Participants and Setting

The BEE scale as well as all other reported measures were designed in the format of an online survey. Participants were undergraduate psychology students from the FernUniversität in Hagen, a distance-teaching university in Germany, who received course credit for participation. The students were informed about the possibility to participate in the study on the website of the department of psychology's “virtual laboratory”. At the time of the study there were more than 10,000 undergraduate psychology students enrolled at the FernUniversität Hagen. However, students are encouraged to get their credits for study participation early in their course of studies. Therefore, only a proportion of students (those at the beginning of their course of studies) might regularly check the website of the “virtual laboratory.” Participation was optional and anonymous. 

### 2.2. Measuring Instruments

#### 2.2.1. Body-Efficacy Expectation (BEE)

The scale was developed according to suggestions based on previously developed domain-specific self-efficacy scales [16]. The items in the scale were also discussed with an experienced expert for self-efficacy in medical psychology. 

The scale consists of six items in the German language. Four items follow the stem sentence “I am sure that my body is well equipped to deal with illness…,” for example, “… even if I sometimes expect too much of it.” Two items follow the stem sentence “I am sure that my body is strong enough to overcome an illness by itself…,” for example, “… even if I am so ill that I have to stay in bed.” Items are answered on a four-point Likert scale ranging from 1 (not at all true) to 4 (exactly true). This answering format corresponds to the answering format suggested by Schwarzer [16] for domain-specific self-efficacy scales as well as to that of the general self-efficacy scale [10]. The mean of all answers is calculated as the final score.

#### 2.2.2. General Self-Efficacy Expectation

General self-efficacy expectation was assessed with the German version of the general self-efficacy scale [10], a widely used and well-validated instrument. The scale assesses the belief that one can cope with demanding and/or novel situations in general, for example, with the item “I am confident that I could deal efficiently with unexpected events.” All ten items are rated on a 4-point-Likert scale ranging from 1 (not at all true) to 4 (exactly true). According to the recommendations by Schwarzer [16] the mean was calculated as the final score if less than 4 items were missing. 

#### 2.2.3. Internal Health Locus of Control (IHLOC)

IHLOC [17–19] was assessed with the subset of seven items referring to internal locus of control from the German version of the multidimensional health locus of control questionnaire [20]. Items refer to the extent one is convinced that oneself is responsible for one's health, for example, “If I take the right actions, I can stay healthy.” They are answered on a 6-point-Likert scale ranging from 1 (strongly agree) to 6 (strongly disagree). Scores are reversed in the process of calculating a final sum score so that a high score represents high internal locus of control. 

#### 2.2.4. Frankfurter Body Concept Scales

The Frankfurter Body Concept Scales [21] assess different body-related self-concepts. We chose three subscales for our study: (1) *state of health (SoH) *assessing the evaluation of one's health or physical limits via 6 items, for example, “I often feel weak,” (2) *body care and external appearance/body functioning (BC/BF)* assessing actions in order to improve physical functioning or appearance via 8 items, for example, “I make sure that I get enough sleep,” and (3) *physical fitness (PF)* assessing evaluations of one's strengths and ductility via 10 items, for example, “I am annoyed by the clumsiness of my movements.” All items are answered on a 6-point-Likert scale ranging from 1 (strongly agree) to 6 (strongly disagree). Negative items are reversed so that high final scores represent a high degree of the respective construct.

#### 2.2.5. Health Status and Chronic Conditions

In addition to the SoH scale, health status was assessed using two questions from the German version of the SF-36 health survey [22]. The item “How would you describe your general health?” assesses general health while the item “How would you describe your present health (compared to last week)?” assesses current health status. The items are answered on a 5-point-Likert scale ranging from “excellent” to “bad” or from “much better than last week” to “much worse than last week,” respectively. The coding was then reversed so that the higher values on the scale indicated better health. 

In addition, we asked if the participants had a chronic condition (yes or no) and if so what chronic condition they had. We also asked for the following acute conditions in the last six months: common cold/flu, stomach problems, headache, back or neck pain, injury, cystitis, vaginal infection (women), psychological problems, and others. If the participants had any of the acute conditions, they were asked to report the duration in days. If the number of days was >100, the condition was considered as chronic and re-classified as such. 

#### 2.2.6. Appraisal of CAM

Participants were asked about their experience with different CAM treatments: homeopathy, TCM, acupuncture, phytotherapy, anthroposophic medicine, body-focused therapies (e.g., yoga, qigong, and taiqi), Bach flowers, essential oils, and up to three other treatments they could specify. They were asked to choose one of the following answer categories: “I have positive experience with this treatment,” “I have negative experience with this treatment,” “I can imagine this treatment is effective, but have not tried it yet,” “I do not think this treatment is effective,” or “I do not know this treatment/no opinion.” The number of treatments that participants reported either to have positive experience with or regarded them as effective even though they themselves had not tried them was used as a surrogate measure for positive CAM appraisal (appraisal score).

#### 2.2.7. Medication Profile

 Participants were asked if they used the following drugs (prescribed or over the counter) during the six months prior to the study: allopathic drugs (e.g., but not limited to pain killers, antibiotics, detumescing nasal spray, beta-blockers, drugs for thyroid dysfunction, and ointments) and psychiatric drugs, together regarded as conventional medicine, phytomedicine, homeopathic drugs, TCM herbs, Bach flowers, and essential oils, together regarded as CAM medicine. They could also use free text to list other medication that afterwards were screened and classified either as conventional or CAM medication. Participants then were assigned to one of the following groups: (1) using only CAM medication, (2) using only conventional medication, (3) using both CAM and conventional medication, and (4) using no medication at all.

#### 2.2.8. Consultation Profile

 Participants were asked if they visited the following physicians or health-care practitioners during the six months before the study: general practitioner or specialist (e.g., but not limited to internists, orthopedic specialist, gynecologist, otorhinolaryngologist, eye specialist, etc.), together regarded as conventional consultations, naturopathic MD, homeopathic MD, TCM MD, anthroposophic medicine MD, and non-medical practitioner (German “Heilpraktiker”), together regarded as CAM consultations. They could also use free text to name other practitioners that afterwards were screened and classified either as conventional or CAM consultation. We also asked for psychotherapy; however, this question was not analyzed. Participants were assigned to one of the following groups: (1) only CAM consultations, (2) only conventional consultations, (3) both CAM and conventional consultations, and (4) not visiting any health-care professional at all.

### 2.3. Statistical Methods and Analyses

#### 2.3.1. Missing BEE Data

BEE items were checked for the amount of missing values. If for one of the items more than 5% of the data were missing, a threshold commonly regarded as crucial [23], Little's MCAR test was conducted in order to check if the data were missing completely at random. If nonsignificant, missing values were imputed using expectation maximization (EM) and imputed values were rounded to integers to reflect the answering format of the scale. All following analyses were conducted with the imputed values. 

#### 2.3.2. Factor Structure and Reliability

We used P-P plots for BEE items to check for normality. If the answers were normally distributed, an exploratory factor analysis (EFA) was conducted using principal axis factoring as extraction method and Promax rotation. Following Hayton, Allen, and Scarpello's tutorial [24], Horn's parallel analysis (PA) was conducted based on 50 randomly created data sets in order to decide on the number of factors and the EFA was then run again with the number of factors fixed to the decided number. Items were screened in order to tag items that warrant consideration for removal. Guiding principles were Costello and Osborne's criteria [25] (i.e., items with communalities <0.40, factor loadings <0.50, or cross-loadings ≥0.32) and other item characteristics as corrected item-(sub)scale correlations, item means, and item variances. At least half of the items in the factors were required to have loadings ≥0.60 to support factor stability [26]. If items were removed, the PA was repeated with the smaller number of items to evaluate if the factor structure still held for the reduced scale. Cronbach's coefficient *α* was calculated for internal consistency, either for the total scale (if of homogeneous factor structure), or for the subscales.

#### 2.3.3. Validity

The health-related scales and questions served as validation measures. P-P plots were used for checking the distribution of continuous measures. If normally distributed, Bravais-Pearson's correlation coefficient *r* was calculated for correlations with BEE. Independent samples *t*-tests were used for comparisons between participants with and without chronic health conditions and between participants with low appraisal and high appraisal of CAM following a split of the sample at the mean of the appraisal score. Regarding the medication profile, a one-factor ANOVA was used to compare BEE between the four groups. Two orthogonal linear contrasts were computed for subgroup analyses in which we compared (1) participants who used no medication at all with all other participants and (2) participants who used CAM medication only with participants who either used only conventional medication or both conventional and CAM medication. A similar analysis was done for the consultation profile with the linear contrasts comparing (1) participants who went to no practitioner at all with all other participants and (2) participants who had CAM consultations only with those who either had conventional only or both CAM and conventional consultations. All CAM-related analyses were repeated separately for participants with and without chronic condition to control for confounding since poorer health status is a predictor for CAM use [27] and might also be related to lower BEE per se. 

All computations were performed using the IBM Statistical Package for the Social Sciences (SPSS), Version 20 for Windows. Since the sample size was large, and therefore statistically significant differences not necessarily are relevant, effect sizes were calculated for *t*-tests and ANOVAs using the program G*Power 3.1 [28]. 

## 3. Results

### 3.1. Participants

1054 students participated in the study. The mean age was 32.74 years (SD = 9.32); 80.4% of the sample were women and 17.9% were men (1.7% did not indicate their gender). 34.8% of the participants had a chronic health condition. 77.3% of the participants had tried a least one CAM treatment. The mean CAM appraisal score was 5.32 (SD = 2.40). 

### 3.2. Missing BEE Data

For the item “… even if I am stressed” 8.5% of the answers were missing; for all other items, the amount of missing values was <1.5%. Little's MCAR test was conducted, yielding a nonsignificant result (*X* ^2^(39) = 36.63, *P* = 0.58) indicating that values were missing completely at random. Missing values then were imputed using the EM algorithm. 

### 3.3. Factor Structure and Reliability

P-P plots suggested that the distributions of answers to the six items were normal. The EFA resulted in a factor solution with two factors having eigenvalues >1.00; however, the PA suggested to just retain the first factor, since it was the only one with an eigenvalue larger than the mean eigenvalue of the EFAs conducted in the 50 random samples (factor 1 : 2.89 versus 1.10; factor 2 : 1.04 versus 1.05). We therefore fixed the number of factors to one and reran the analysis for the interpretation of item parameters. One item—“... even if I do not take many precautions (e.g., wash hands regularly, wear a face mask, prevent contact with people who are contagious, flu vaccine, dietary supplements)”—showed very low communality (<0.30) as well as a much lower corrected item-total correlation and higher standard deviation than the other items. Since it could be justified with regard to the content to drop this item, we decided to run an EFA with the remaining five items instead. Again, the PA suggested to just retain one factor (factor 1: 2.60 versus 1.09; factor 2: 1.02 versus 1.04). The number of factors was thus fixed to one. The resulting solution explained 51.96% of the variance, and the factor loadings were sufficiently high (four items >0.60). However, three items showed communalities <0.40. 

Cronbach's alpha for the 5-item scale was *α* = 0.77. Omitting any item from the scale would not lead to an increase in *α*. The average inter-item-correlation was *r* = 0.40 (0.25–0.66).  Table 1 shows item means, factor loadings, corrected item-total correlations, and communalities for all items. 

### 3.4. Validity

The BEE mean score was *M* = 2.83 (SD = 0.54). This is comparable to other domain-specific self-efficacy scales [7] (see Table 2 for descriptive statistics and internal consistency of all validation measures). The P-P plots indicated that the distributions of the validation measures were normal. Table 2 shows the intercorrelations of all scales. Positive correlations were found between BEE and general health (no matter if assessed with the SoH scale or the item of the SF-36), fitness, general self-efficacy expectations, and IHLOC with the highest correlations being those between BEE and the general health measures. No correlations were found between BEE and current health status as well as body care. 

Participants suffering from a chronic condition had significantly lower BEE than participants without chronic conditions (*t*(1033) = 7.09; *P* < 0.001; effect size *d* = 0.46), and participants with high appraisal of CAM had higher BEE than those with low appraisal of CAM in the total sample (*t*(1052) = 4.04; *P* < 0.0001; effect size *d* = 0.25) as well as in the subgroups of participants with chronic condition (*t*(365) = 3.07; *P* < 0.01; *d* = 0.32) and no chronic condition (*t*(666) = 2.97; *P* < 0.01; *d* = 23). The results of the ANOVAs are shown in Tables 3 and 4. No significant mean differences were found for the subgroup suffering from a chronic condition, but for both the total sample and the healthy subgroup. The linear contrasts showed that participants who took no medication at all had higher BEE than those who took any medication, and participants who took only CAM medication had higher BEE than those taking either only conventional or both CAM and conventional medication (Table 5). Regarding the consultation profiles, the only significant mean differences were those between participants who consulted no health care professional at all and those who did in both the total sample and the subgroup without chronic condition (Table 6). 

## 4. Discussion

The purpose of this study was to develop and validate a scale to measure BEE, that is, the conviction that one's body is able to deal with pathogens and other health-threatening demands on its own. The development of the scale followed the line of domain-specific self-efficacy research; however, the concept deviates from other specific self-efficacy scales as the behavior of the body is not actively executable. 

The process of identifying the factor structure and deciding on the final items proved rather complex. One of the initially six items did not meet several criteria for a satisfactory factor solution. With regards to content, the item was different from the remaining items insofar that “normal” behavior (not wearing a mask, not avoiding ill people, and not washing your hands often) was regarded as a potential health risk. Therefore, a low score for this item might have indicated if someone had tendencies of an illness phobia or obsessive-compulsive disorder with contamination concerns [29, 30] rather than low BEE. For this reason we considered it appropriate to remove the item from the scale and in doing so improve the factor structure and make the scale more straightforward. The results of the EFA with PA suggested a one-factor solution for the remaining five items. However, the communalities did not all reach the threshold of .40, indicating that not all variables are highly related to each other. This reflects the very tight decision for a one-factor solution, with the eigenvalue from the EFA's second factor being just below the mean of eigenvalues of the PA. A two-factor structure reflecting the two different stem sentences of the scale and therefore convictions about the body's capability to stay healthy and to get healthy when already ill, however, would have resulted in one of the factors consisting of only two items. Two-item factors are generally considered weak and unstable while factors with “5 or more strongly loading items (0.50 or better) are desirable and indicate a solid factor” [25]. Since all five items had factor loadings >0.50, and Cronbach's *α* still was sufficiently high [31] the one-factor solution probably is the best despite the rather low communalities. 

The scale's correlations with the validation measures were in line with previous research and most important with those considerations on the formation and possible effect of BEE.

The positive relationship between BEE and IHLOC suggests an overall feeling of empowerment (or, resp., helplessness) regarding one's own health, either through one's own behavior (as is assessed by IHLOC) or through trust in one's body's capability (as is assessed by the BEE scale). This is not in line with Luszczynska and Schwarzer [32] who argue that correlations between health locus of control and specific self-efficacy measures tend to be low because locus of control refers to a broader range of health beliefs than domain-specific self-efficacy scales. BEE, however, also is rather unspecific regarding the health problems it targets which might well explain the positive correlations found in our study. In fact, correlations between domain-specific self-efficacy expectations and IHLOC have been found in other studies as well [33, 34].

Feeling empowered in the health domain furthermore relates to a general trust in one's ability to deal with problems as the correlation between BEE and general self-efficacy suggests. Such moderate correlations between domain-specific and general self-efficacy have been reported for other domain-specific self-efficacy scales as well [10, 12]. 

Most important, participants with a better general health status (irrespective of whether the assessment was with the SoH scale or with the item from the SF-36) reported higher BEE. Also, participants without chronic condition had higher BEE than those with chronic condition. In the total sample and in the subgroup without chronic condition, those participants who took no medication at all had higher BEE than those who did, and those who did not consult any health care professional had higher BEE than those who did. Therefore, the experience that the body is healthy in general is paired with a certain trust in its abilities. Since the study followed a cross-sectional design, the nature of this relationship cannot be clarified. However, as outlined in the introduction section, influences possibly are bidirectional. Performance accomplishments, in this case being generally of good health, can boost self-efficacy expectations [6]. However, higher BEE might also lead to a better health. This is suggested by the placebo research as well as by studies that show effects of self-efficacy expectations on immune system parameters such as cortisol levels or numbers of lymphocytes [35]. 

Present health status, on the other hand, did not correlate with BEE. This is in line with Bandura [6]. Although self-efficacy expectations are modifiable, adjustments are not made quickly (i.e., by experiencing a better or worse health than in the previous week) and changes are reliant on strong new experiences. 

Also, no correlation was found between BC/BF and BEE. The BC/BF items deal with concerns as getting enough sleep, keeping up dental care, eating healthy, or with general convictions such as “a healthy mind belongs to a healthy body.” It is possible that high expectations regarding one's body's capabilities may actually minimize the importance of such concerns (if someone feels that his/her body is highly capable, it might not even be important to get enough sleep). 

Physical fitness, on the other hand, was related to BEE. Fitness in fact correlates with better health and a more positive attitude towards one's body (lower levels of physical activity have been noted in subjects with hypochondria or illness worries [36]); however, in our study the relation might mostly be due to the substantial correlation between physical fitness and state of health. 

High appraisal of CAM was associated with higher BEE. Also, higher BEE was found in participants who took only CAM medication compared to those who took only conventional or both CAM and conventional medication, as long as they did not suffer from a chronic condition. Again, the direction of influence cannot be concluded. As outlined in the introduction section, self-healing beliefs can be a result of CAM treatment [15] as patients adopt concepts as “balance,” “qi,” or “energy,” from their practitioner [[14], page 564]. Concerning other kinds of domain-specific self-efficacy expectations, it has indeed been shown that it is possible to enhance them by appropriate interventions [37–42]. However, it is also assumed that people chose CAM because it is in accordance with beliefs that they already hold [43, 44]. In this scenario BEE would not be a result of but reason for CAM use. Possibly both directions of impact play together (resulting in a self-stabilizing or destabilizing system of facilitated/complicated healing and higher/lower BEE). Longitudinal studies and structural equation modelling might be useful to further disentangle the mutual influences in future research. 

Several strengths of the study can be highlighted. (1) The sample was large and quite heterogeneous. Participants were students of the only distance-teaching university in Germany who vary considerably in age and in their lifestyle as well as in previous knowledge and experience. Unlike in German on-site universities, there is no grade point enrolment limit for this university. Many of the students have work experience; 80% work during their course of studies [45], and many have children. The proportion of participants with a chronic condition was comparable to the German population [46]. However, the majority of the samples were women who differ from men in their CAM use [47] and also in their health beliefs and behavior [48]. (2) We used a large set of validation measures to embed BEE in a broad context. (3) Regarding the CAM-related variables (appraisal of CAM, medication profile, and consultation profile), we divided the sample in subgroups with and without chronic condition to control for confounding. In fact, most results were not significant in participants suffering from a chronic condition indicating that this in fact is an important confounder. 

Several limitations have to be discussed. (1) The scale was developed just by following recommendations and not by starting with a large item pool and reducing it in several steps. However, the recommendations were drawn up by a well-recognized expert in self-efficacy research. (2) Communalities for some items were below the common threshold of .40 indicating a trend towards a heterogeneous factor structure. A clearer factor structure might have been achieved by formulating more items and thereby strengthening a possible second factor or by limiting the scale items to one of the domains indicated by the two stem sentences (staying healthy or getting healthy when already ill). However, as the factor loadings and Cronbach's *α* are still sufficiently high, the current scale might be an economical measure for the quite broad construct of BEE inclusive of both its aspects. (3) Health status and chronic conditions were only assessed via self-report. Analyzing samples with a confirmed diagnosis, for example, chronic pain, might be worthwhile. (4) The CAM appraisal score might not be the most appropriate measure to assess the appraisal of CAM. Someone who is strongly convinced of one kind of CAM, for example, homeopathy, might stick to that one treatment only and not believe in anything else. Such a person in our study would fall into the low appraisal group while someone who has tried one or the other alternative treatments and found them working alright but might be convinced otherwise in a twinkling would fall into the high appraisal group. The same is true for the medication and consultation profiles that might simplify someone's health-related behavior in a nonrepresentative manner. (5) In the ANOVAs and contrast analyses, the compared groups were of different sample size. The same was true for the subgroups of participants with and without chronic condition. However, this cannot be avoided in observational studies. (6) An alpha of 0.5 was assumed for all statistical tests. Therefore testing was multiple and results should be interpreted with some caution regarding statistical significance. (7) Regarding the CAM-related analyses (BEE's relations with CAM appraisal, medication score, and consultation score) most absolute differences and effects were rather small with effect sizes of about 0.2 that correspond to small effects [49]. 

## 5. Conclusion

Since arriving at the final factor structure and items involved rather tight thresholds and various considerations, further demonstrations that the factor structure is reliable are recommended, for example, via confirmatory factor analyses in other large samples. Also, more research on BEE's relation with CAM and directions of influence is needed. However, since the scale substantially correlated with the validation measures in the assumed direction, in the meantime we consider the scale applicable in CAM as well as in health psychology as it adds a new component to expectancy research that is especially relevant to CAM. 

## Figures and Tables

**Table 1:** Factor loadings and item parameters of the BEE scale.

	Item mean (SD^a^)	Factor loading	Corrected item-total-correlation	Communality
I am sure my body is well prepared for illness…				
... even if I sometimes expect too much of it.	2.91 (0.74)	0.68	0.59	0.47
... even if I am exposed to a lot of pathogens in public.	2.98 (0.78)	0.57	0.49	0.32
... even if I am stressed.	2.58 (0.76)	0.61	0.52	0.37
I am sure my body is strong enough to overcome an illness by itself…				
... even if I am so ill that I have to stay in bed.	3.01 (0.71)	0.68	0.57	0.46
... even if I have an illness over a longer period.	2.65 (0.74)	0.62	0.51	0.38

^a^Standard deviation.

Excluded item:... even if I do not take many precautions (e.g., wash hands regularly, wear a face mask, prevent contact with people who are contagious, get a flu vaccine, or take dietary supplements).

**Table 2:** Descriptive statistics, internal consistencies, and intercorrelation of the scales.

	BEE	GSE	IHLOC	SoH	BC/BF	PF	General Health	Present Health
Intercorrelation of the scales								
BEE: body-efficacy expectation	—							
GSE: general self-efficacy	0.35	—						
IHLOC: internal health locus of control	0.30	0.23	—					
SoH: state of health	0.47	0.43	0.29	—				
BC/BF: body care and external appearance/body functioning	0.09	0.21	0.31	0.27	—			
PF: physical fitness	0.30	0.34	0.23	0.68	0.42	—		
General health	0.47	0.31	0.28	0.65	0.23	0.45	—	
Present health	−0.04	−0.01	0.00	−0.03	−0.03	−0.04	−0.03	—

Mean (M)	2.83	2.97	29.34	25.70	35.70	44.04	3.35	3.19
Standard Deviation (SD)	0.54	0.43	4.93	5.30	5.18	8.59	0.81	0.75
Cronbach's *α*	0.77	0.88	0.84	0.84	0.73	0.87		

**Table 3:** ANOVAs: body-efficacy expectation compared between different medication profiles.

	Total group		No chronic condition		Chronic condition	
	*N*	Mean (SD)	*n*	Mean (SD)	*n*	Mean (SD)
CAM medication only	100	2.97 (0.48)	77	3.02 (0.47)	23	2.77 (0.48)
Conventional medication only	350	2.79 (0.55)	202	2.89 (0.52)	147	2.67 (0.57)
Both	320	2.71 (0.52)	162	2.79 (0.49)	156	2.63 (0.55)
No medication	284	2.96 (0.51)	227	2.99 (0.50)	41	2.82 (0.49)

Total	1054	2.83 (0.54)	668	2.91 (0.51)	367	2.67 (0.55)

*F* (df)		14.06 (3,1050)		6.40 (3,664)		1.61 (3,363)
*P*		<0.001		<0.001		0.19
Effect size *f*		0.20		0.17		0.11

**Table 4:** ANOVAs: body efficacy-expectation compared between different consultation profiles.

	Total group		No chronic condition		Chronic condition	
	*N*	Mean (SD)	*n*	Mean (SD)	*n*	Mean (SD)
CAM consultations only	25	2.83 (0.45)	17	2.82 (0.44)	8	2.85 (0.49)
Conventional consultations only	531	2.74 (0.54)	294	2.81 (0.51)	235	2.65 (0.57)
Both	80	2.70 (0.53)	35	2.89 (0.46)	44	2.57 (0.54)
No consultations	418	2.96 (0.51)	322	3.02 (0.50)	80	2.78 (0.48)

Total	1054	2.83 (0.54)	668	2.91 (0.51)	367	2.67 (0.55)

*F* (df)		16.24 (3,1050)		9.11 (3,664)		1.91 (3,363)
*P*		<0.001		<0.001		0.13
Effect size *f*		0.21		0.20		0.13

**Table 5:** Linear contrasts for comparisons between medication profiles.

	Total group			No chronic condition			Chronic condition		
	Difference in BEE	*t* (df)	*P*	Difference in BEE	*t* (df)	*P*	Difference in BEE	*t* (df)	*P*
No medication versus any medication	0.41	3.52 (1050)	<0.001	0.27	2.12 (664)	<0.05	0.39	1.36 (363)	0.18
CAM medication only versus conventional or both CAM and conventional medication	0.43	3.84 (1050)	<0.001	0.37	2.94 (664)	<0.01	0.26	1.08 (363)	0.28

BEE: body-efficacy expectation.

**Table 6:** Linear contrasts for comparisons between consultation profiles.

	Total group			No chronic condition			Chronic condition		
	Difference in BEE	*t* (df)	*P*	Difference in BEE	*t* (df)	*P*	Difference in BEE	*t* (df)	*P*
No consultation versus any consultation	0.62	4.30 (1050)	<0.001	0.53	3.10 (664)	<0.01	0.26	0.93 (363)	0.35
CAM consultation only versus conventional or both CAM and conventional consultation	0.23	1.04 (1050)	0.30	−0.05	−0.19 (664)	0.85	0.48	1.21 (363)	0.23

BEE: body-efficacy expectation.
